# Assessment of nucleic acid extraction protocols for antibiotic resistance genes (ARGs) quantification in aircraft wastewater

**DOI:** 10.1186/s40246-024-00617-5

**Published:** 2024-05-30

**Authors:** Wendy J. M. Smith, Yawen Liu, Stuart L. Simpson, Aaron Bivins, Warish Ahmed

**Affiliations:** 1 CSIRO Environment, Ecosciences Precinct, 41 Boggo Road, Dutton Park, QLD 4102 Australia; 2 State Key Laboratory of Marine Environmental Science, College of the Environment and Ecology, Xiamen University, Xiamen, 361102 China; 3 Department of Civil and Environmental Engineering, Louisiana State University, Baton Rouge, LA 70803 USA

**Keywords:** Transportation, Importation, Surveillance, AMR, ARG, ARB

## Abstract

This study evaluated ten nucleic acid extraction protocols (EP1 to EP10) for measuring five endogenous antibiotic resistance genes (ARGs) in four aircraft wastewater samples (AWW1 to AWW4). The targeted ARGs, including *bla*_*CTX-M*_, *bla*_*NDM-1*_, *ermB*, *qnrS*, and *tetA*, encompassed highly and minimally abundant ARGs. *TetA* and *ermB* were consistently detected across four aircraft wastewater samples using the DNeasy Blood and Tissue Kit and the AllPrep PowerViral DNA/RNA kit. *QnrS* displayed high detection rates with specific extraction protocols and aliquot volumes. Concentrations of ARGs varied across aircraft wastewater samples, with differing extraction protocols influencing quantitative results. The concentrations of *tetA*, *ermB*, and *qnrS* in AWW1 were distinct, while AWW2 to AWW4 exhibited a broader range for *tetA*, *ermB*, *qnrS*, *bla*_*CTX-M*_, and *bla*_*NDM-1*_. EP1 consistently produced the highest concentrations for several ARGs. Collective data analysis revealed varying ARG concentrations across the ten extraction protocols, suggesting the importance of careful extraction protocol selection in ARG monitoring in aircraft wastewater samples. Based on the results, we suggest that a small sample volume (as low as 0.2 mL) may be sufficient for ARG characterization in aircraft wastewater samples. The findings also emphasize the need for considering toilet paper removal without compromising nucleic acid extraction efficiency. The study highlights promising prospects for aircraft wastewater monitoring of ARGs, calling for further investigation into the import and spread of unique ARGs through transport hubs.

**Supplementary Information:**

The online version contains supplementary material available at 10.1186/s40246-024-00617-5.

## Introduction

Antimicrobial resistance (AMR) has profound implications for the efficacy of clinical treatments for infectious diseases and stands out as a critical concern, ranking among the top ten threats to global health [33]. The spread of AMR is driven by a complex web of factors that disseminate antibiotic resistance genes (ARGs) among bacteria within the interconnected realms of the environment, animals, and humans [16]. Among these realms, the environment plays a pivotal role in the emergence and dissemination of ARGs, as well as potential human exposures to antibiotic-resistant bacteria (ARB) [21].

Global travel facilitates the worldwide spread of human pathogens, given that the human body serves as an ideal host for numerous infectious agents such as respiratory and enteric viruses, pathogenic bacteria, and multi-drug resistant bacteria and fungi [20]. An important pathway for ARG dissemination is the increasing interconnection of disparate human populations via passenger air travel [13]. Prior to the COVID-19 pandemic, commercial airlines were transporting up to 4.5 billion passengers on as many as 38.6 million global flights. Although flight numbers decreased significantly during the pandemic (16.8 million in 2020), by 2022, passenger numbers had recovered to 3.8 billion, and by August 2023, the numbers had reached 95.7% of pre-pandemic levels [16]. Since air travel is a relevant mechanism for the global spread of AMR, unintrusive surveillance of ARBs and ARGs among passengers is a strategic opportunity and one that has been previously explored via aircraft wastewater.

In 2015, Danish scientists conducted a metagenomic analysis of aircraft wastewater collected from long-distance flights to assess global patterns of AMR and enteric pathogens [24]. The researchers used shotgun sequencing to analyze toilet waste collected from 18 international flights arriving in Copenhagen, Denmark, originating from nine cities across three distinct regions (North America, North Asia, and South Asia). Wastewater pellet was purified using a protocol including both lysozyme and lysostaphin to increase cell lysis followed by phenol/chloroform extraction. Their analysis revealed variations in the abundance and diversity of ARGs and the abundance of certain enteric pathogens based on the flight's region of origin. This pioneering study underscored the potential utility of aircraft wastewater as a valuable tool in the global battle against infectious diseases and antimicrobial resistance.

In another study, a combination of shotgun sequencing, quantitative polymerase chain reaction (qPCR), and culture-based methods were employed to investigate the presence of ARBs and ARGs in aircraft wastewater [15]. Samples were collected from lavatory service trucks, which contained wastewater from multiple international flights arriving at five German airports. DNA was extracted using PowerWater Kit (MoBio, Vancouver, Canada). The study found an increased relative abundance of ARGs in aircraft wastewater samples compared to municipal wastewater from the respective airport cities. Simultaneously, *E. coli* isolates from aircraft wastewater samples were resistant to multiple drugs at eight times greater prevalence than clinical isolates from Germany. These results suggest that aircraft wastewater could be a significant reservoir for antibiotic resistance, potentially contributing to the global spread of ARBs and their associated genes between countries.

Dissemination of AMR via wastewater (aircraft and/or municipal) is multi-dimensional, involving both horizontal and vertical transfers of genetic material via replication-competent cells. As such, various microbiology techniques, including culture-based methods, qPCR, and metagenomics, have proven useful for studying ARBs and ARGs in wastewater. The abundance and diversity of ARGs in wastewater is influenced by a variety of factors, including their host sources, initial concentrations, the location of ARGs on chromosomes or mobile genetic elements (MGEs), and their ability to persist in the environment [4, 5]. Notably, qPCR-based approaches have gained popularity for their high efficiency, sensitivity, and specificity for measuring mobile genetic elements and ARGs in aquatic environments. These qPCR assays can precisely quantify gene abundance, facilitating the assessment of ARG prevalence and dynamics in wastewater and other water samples. Moreover, qPCR can differentiate between different ARGs, making it a valuable tool for monitoring specific resistance genes, especially at low abundance.

Whether for qPCR or sequencing, molecular endpoint workflows often include sample concentration and nucleic acid extraction steps [9, 21]. The design and performance characteristics of these multi-step workflows must be carefully chosen considering the decisions to be made with the data. In a recent study, we compared the estimated concentrations of endogenous 16S ribosomal (16S rRNA) gene of bacteria, mobile genetic element *intI*1, and ARG *van*A in municipal wastewater samples varying several key methodological parameters, including the sample volume, membrane types, and extraction kits [22]. We found a small sample volume (down to 2 mL) was sufficient for consistent detection of highly abundant ARGs. However, little is known regarding the impacts of various nucleic acid extraction protocols for ARG quantification in aircraft wastewater samples.

In this study, various nucleic acid extraction protocols were compared for the measurement of five endogenous ARGs including *bla*_*CTX-M*_ (encoding extended-spectrum β-lactamase enzyme), *bla*_*NDM-1*_ (encoding the New Deihi metallo-β-lactamase-1 enzyme), *ermB* (conferring resistance to erythromycin), *qnrS* (conferring resistance to fluoroquinolone) and *tetA* (conferring resistance to tetracycline) in aircraft wastewater samples. The rationale for selecting these genetic targets is that they encompass both highly and minimally abundant ARGs, including genes located on chromosomes as well as those associated with MGEs. The results of this study will be useful to inform the selection of an optimal workflow for the detection and quantification of low levels (near the assay detection limit) of ARGs in aircraft wastewater samples with high sensitivity and reproducibility.

## Materials and methods

### Sample preparation and nucleic acid extraction protocols

**Fig. 1:**
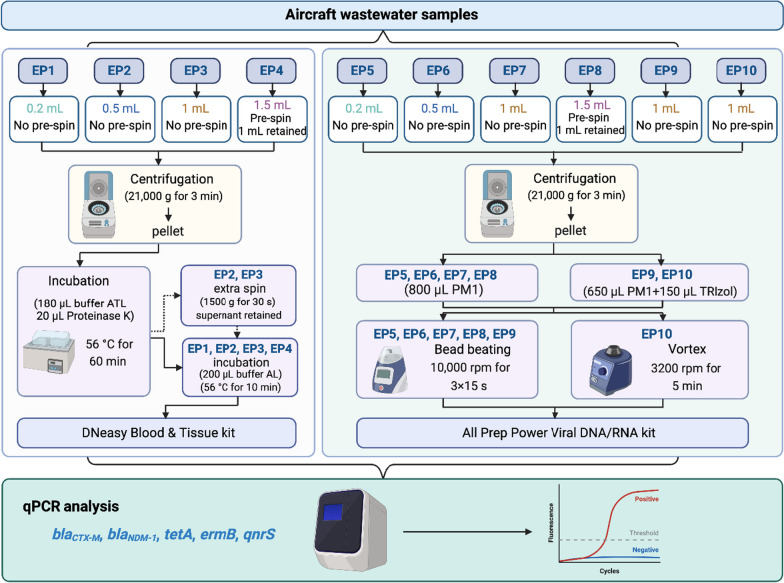
Extraction protocols (EP1 to EP10) used in this study for qPCR detection and quantification of *tetA*, *ermB*, *qnrS*, *bla*_*CTX-M*_ and *bla*_*NDM-1*_ in aircraft wastewater samples

Four archived aircraft lavatory wastewater samples (− 20 °C) were thawed at 4 °C overnight, denoted as AWW1, 2, 3 and 4. Wastewater samples were subjected to nucleic acid extraction using the DNeasy Blood and Tissue Kit (Cat. No. 69506) (Qiagen, Hilden, Germany) and AllPrep PowerViral DNA/RNA Kit (Cat No. 28000-50) (Qiagen) (Supplementary Table ST1). These two kits were specifically chosen to accommodate small volume (i.e., < 1.5 mL) wastewater samples. For the DNeasy Blood and Tissue Kit, four different starting aliquots of wastewater (0.2 mL, 0.5 mL, 1 mL, and 1.5 mL) were used. The 1.5 mL aliquot (EP4) was centrifuged slowly at 1500 g for 30 s to pellet any toilet paper, and then 1 mL of the resulting supernatant was transferred to a sterile 2 mL tube. All (EP1 to EP4) wastewater samples were then centrifuged at 21,000 g for 3 min, and the supernatant was discarded, leaving the final pellets for extraction (Fig. 1). Nucleic acids were extracted from the EP1 to EP4 pellets. Briefly, each of the pellets was resuspended in 180 µL of ATL buffer, 20 µL of proteinase K was added, mixed thoroughly, and then incubated at 56 °C for 60 min. In the next step, the EP2 and EP3 tubes were centrifuged slowly at 1500 g for 30 s and the supernatant was transferred into 2 mL tubes leaving undigested toilet paper behind. Then 200 μL of buffer AL was added into each EP1 to EP4 tubes and incubated at 56 °C for 10 min. Finally, 200 μL of ethanol was added, and then the resulting solution was loaded onto the spin columns, and nucleic acid was extracted as per the manufacturer's instructions.

For the AllPrep PowerViral DNA/RNA Kit (Qiagen), four different starting aliquots of wastewater (0.2 mL, 0.5 mL, 1 mL, and 1.5 mL) were also used for aliquots EP5 to EP10. The 1.5 mL aliquot (EP8) was centrifuged slowly at 1500 g for 30 s to pellet any toilet paper, and then 1 mL of the resulting supernatant was transferred to a sterile 2 mL tube. All (EP5 and EP10) wastewater samples were then centrifuged at 21,000 g for 3 min, and the supernatant was discarded, leaving the final pellets for extraction. For aliquots EP5 to EP8, pellets were lysed using 800 µL of buffer PM1 and 8 µL β-Mercaptoethanol (Cat. No. M6250-10 mL) (Sigma-Aldrich, St. Louis, Missouri, USA).

For the EP9 and EP10 pellets (after centrifugation), 650 µL of PM1, 8 µL β-Mercaptoethanol and 150 µL of Trizol® reagent (Ambion, Sigma-Aldrich, California, USA) were added for lysis. During lysis, the AllPrep aliquots (EP5 to EP9) were homogenized using a Precellys 24 tissue homogenizer (Bertin Technologies, Montigny-le-Bretonneux, France) set for 3 × 15 s at 10,000 rpm at 10 s intervals. EP10 was vortexed for 5 min at 3200 rpm on the Vortex-Genie®-2 (Scientific Industries) in the vertical holder (Cat. No.146-6005-00). After homogenization, nucleic acid extraction was completed as per the Qiagen AllPrep PowerViral DNA/RNA Kit (Qiagen) instructions except elution was performed with 200 µL of RNase-free water to match the elution volume of the DNeasy Blood and Tissue Kit (Qiagen). The concentrations of extracted DNA were determined by a DeNovix Spectrophotometer/Fluorometer (DeNovix DS-11, Wilmington, USA). 

### qPCR analysis

The qPCR primers, probes and cycling parameters for assays targeting *bla*_*CTX-M*_, *bla*_*NDM-1,*_* ermB, qnrS* and *tetA* are provided in Supplementary Table ST2. All qPCR amplifications were performed in 20 μL reaction mixtures using 2 × QuantiNova Probe PCR Master Mix (Qiagen). The qPCR mixtures contained 10 μL of Master Mix, 300 to 1000 nM of forward primer, 300 to 1000 nM of reverse primer, 100 to 200 nM of probe, and 3 μL of nucleic acid template. A series of gBlock gene fragment standards (Integrated DNA Technologies, Coralville, IA, USA) ranging from 3 × 10^6^ to 3 GC/reaction were used to create calibration curves to quantify target genes. All qPCR reactions were performed in triplicate with negative controls included in each qPCR run. All qPCR experiments were performed using a Bio-Rad CFX96 thermal cycler (Bio-Rad, California, USA). The threshold and baseline were all adjusted to values ranging from 30 to 100 RFU for each qPCR assay.

### Quality control

Quality assurance/control metrics for qPCR calibration curves including efficiencies, coefficients of determination (r^2^), and y-intercepts were documented per the Minimum Information for Publication of Quantitative Real-Time PCR Experiments (MIQE) guidelines [8]. A Sketa22 real-time PCR assay was applied to assess PCR inhibition by seeding a known copy number (10^4^ gene copies (GC)) of *Oncorhynchus keta* (*O. keta*) DNA in extracted DNA samples [14]. The reference quantification cycle (Cq) value was determined from triplicate reactions containing only the positive control. The mean reference Cq value was compared with the Cq values of all samples. If the Cq values of nucleic acid samples were within 2-Cq values of the reference Cq value the sample was considered to have no PCR inhibition [29]. Inhibited nucleic acid samples were diluted to fivefold or tenfold and reanalyzed. Nucleic acid extraction and qPCR setup were performed in separate laboratories to minimize the potential for cross contamination during experiments.

### Statistical analysis

Samples were classified as positive if the qPCR amplification was observed in at least two out of three replicates within 45 cycles. Samples were classified as quantifiable if the qPCR amplification was observed in three out of three replicates and within 40 cycles. GC concentrations were log_10_ transformed and expressed as log_10_ GC/mL of aircraft wastewater. All data were tested for normality and homogeneity of variances using GraphPad software (Prism 9.5.1, La Jolla, CA, USA). The log_10_ GC/mL concentrations obtained from each aircraft wastewater sample were pooled and analyzed for ARGs both individually and collectively. This analysis was conducted using repeated measures one-way analysis of variance (ANOVA). If the concentration data did not follow a normal distribution, a Friedman test was used instead [26].

## Results

### DNA quantity, quality, and qPCR performance characteristics

Concentration (ng/μL) of DNA and absorbance (*A*_260/280_) values for aircraft wastewater samples extracted using ten protocols are shown in Supplementary Table ST3. The qPCR standard curves for all five ARG targets (*tetA*, *ermB*, *qnrS*, *bla*_*CTX-M*_, *bla*_*NDM-1*_) showed a dynamic linear range of quantification from 3 × 10^6^ to 3 GC/reaction (1 × 10^6^ to 1 GC/μL). The slopes were − 3.51 (*tetA*), − 3.38 (*ermB*), − 3.10 (*qnrS*), − 3.55 (*bla*_*CTX-M*_), − 3.58 (*bla*_*NDM-1*_), and the amplification efficiencies were 92.8%, 97.7%, 110%, 91.3% and 90.1%, respectively (Supplementary Table ST4). The Y-intercepts were 39.8 (*tetA*), 38.8 (*ermB*), 41.8 (*qnrS*), 45.9 (*bla*_*CTX-M*_), 40.5 (*bla*_*NDM-1*_) and the coefficients of determination (*r*^2^) ranged from 0.94 to 1.00. All DNA samples were within the 2-Cq values of the reference Cq value except for aircraft wastewater samples AWW1 (EP9 and EP10) and AWW2 (EP9), which all employed the AllPrep PowerViral DNA/RNA Kit with PM1 + TRIzol for lysis.

### Detection rates of *tetA***, ***ermB***, ***qnrS*, *bla*_*CTX-M*_ and *bla*_*NDM-1*_ in aircraft wastewater samples

*TetA* and *ermB* were detected in all aircraft wastewater samples extracted using the DNeasy Blood and Tissue Kit (EP1 to EP4) and the 1.0 mL and 1.5 mL aliquots extracted via AllPrep Power Viral DNA/RNA kit (EP7 and EP8). Additionally, *ermB* had a 100% detection rate in the 0.5 mL aliquot extracted via AllPrep Power Viral DNA/RNA kit (EP6). For *qnrS,* 100% detection rates were observed in the 0.2 mL, 1.0 mL and 1.5 mL aliquots extracted via the DNeasy Blood and Tissue Kit (EP1, EP3, EP4) (Table 1). Consistent detection rates (3/4; 75%) were observed in three of four aircraft wastewater samples for *bla*_*CTX-M*_ using all extraction protocols and aliquot volumes. The less-frequently observed, *bla*_*NDM-1*_ was mainly detected (0–75%) in aircraft wastewater samples using the 0.5 mL aliquot extracted via the DNeasy Blood and Tissue Kit (EP2) (three of four aircraft wastewater samples), followed by extraction protocols using the 0.5 mL and 1.0 mL aliquots extracted via AllPrep PowerViral DNA/RNA Kit (EP6 and EP7) (two of four aircraft wastewater samples). Overall, ARGs were less frequently observed in AWW1, and *tetA, ermB, qnrS, bla*_*CTX-M*_ were detected in AWW2-AWW4 using all sample aliquot volumes with all extraction protocols.

**Table 1:** Detection rates of ARGs in aircraft wastewater samples using different extraction protocols

ARGs	Extraction protocols (%)										Aircraft wastewater samples (%)			
	EP1	EP2	EP3	EP4	EP5	EP6	EP7	EP8	EP9	EP10	AWW1	AWW2	AWW3	AWW4
*tetA*	100	100	100	100	75	75	100	100	75	75	60	100	100	100
*bla*_*CTX-M*_	75	75	75	75	75	75	75	75	75	75	0	100	100	100
*bla*_*NDM-1*_	25	75	0	25	25	50	50	25	0	0	0	70	20	20
*ermB*	100	100	100	100	75	100	100	100	75	75	70	100	100	100
*qnrS*	100	75	100	100	75	75	75	75	75	75	30	100	100	100

### Concentrations of *tetA*, *ermB*, *qnrS*, *bla*_*CTX-M*_ and *bla*_*NDM-1*_ in aircraft wastewater samples

**Fig. 2:**
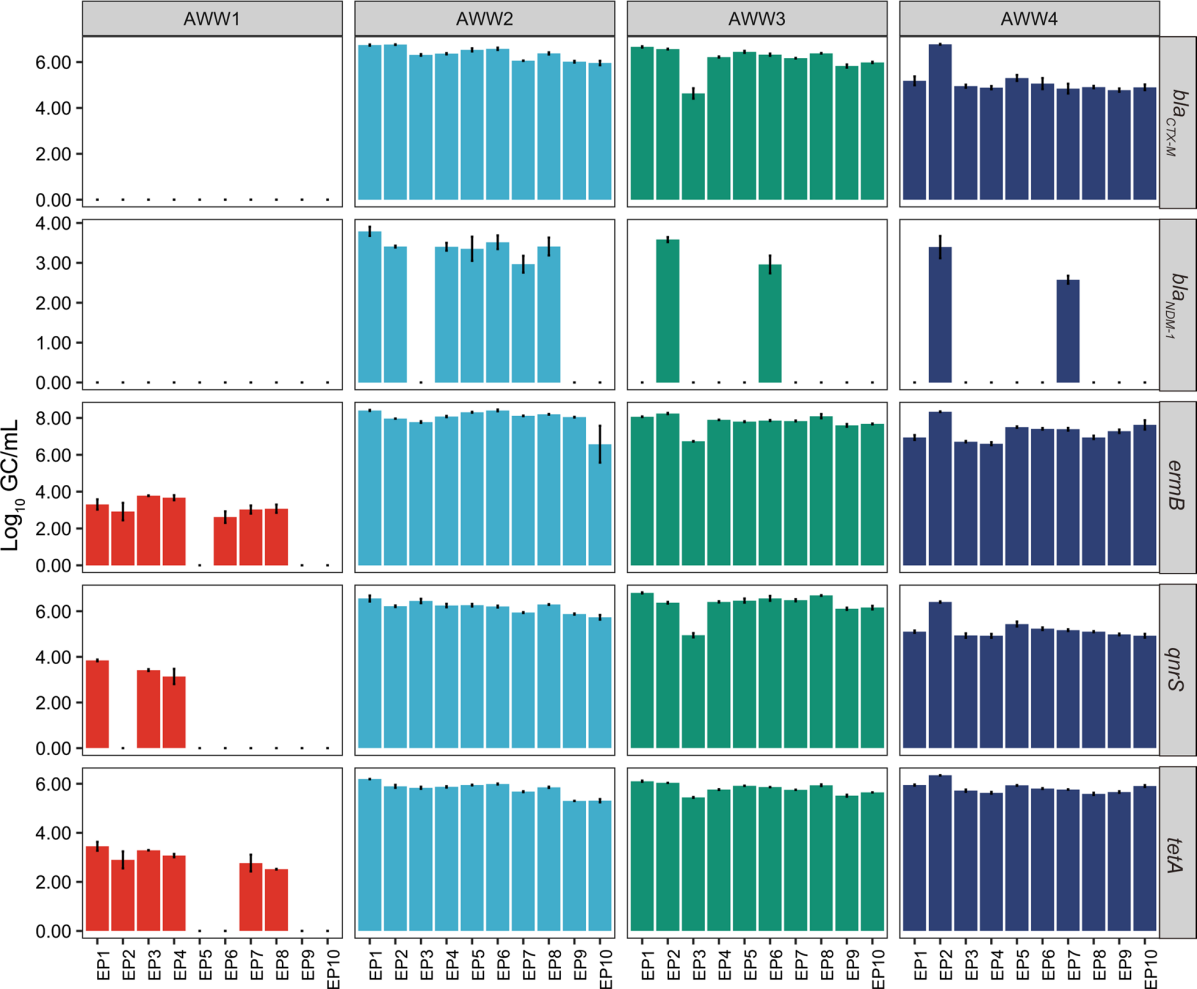
Mean concentrations (log_10_ GC/mL) of *tetA*, *ermB*, *qnrS*, *bla*_*CTX-M*_ and *bla*_*NDM-1*_ in aircraft wastewater samples using 10 extraction protocols. Error bars represent the standard deviation for qPCR triplicates

The concentrations of ARGs exhibited variations among aircraft wastewater samples using different extraction protocols. In AWW1, the concentration of *tetA*, *ermB*, *qnrS* ranged from 3.45 log_10_ GC/mL for *tetA*, 3.67 log_10_ GC/mL for *ermB*, 3.84 log_10_ GC/mL for *qnrS* to non-detection (Fig. 2 and Supplementary Table ST5). The greatest concentrations of *tetA*, *ermB* and *qnrS* in AWW1 were obtained from the 0.2 mL aliquot extracted via the DNeasy Blood and Tissue Kit (EP1), 1.5 mL pre-spin aliquot extracted via DNeasy Blood and Tissue Kit (EP4), and EP1, respectively. In samples AWW2-AWW4, the concentrations of *tetA*, *ermB*, *qnrS*, *bla*_*CTX-M*_ ranged from 5.30 to 6.34 log_10_ GC/mL, 6.59 to 8.41 log_10_ GC/mL, 4.92 to 6.80 log_10_ GC/mL and 4.63 to 6.77 log_10_ GC/mL, respectively. Among which, EP1 generated the highest concentrations of *tetA*, *ermB*, *qnrS* and *bla*_*NDM-1*_ in AWW2 and the highest concentrations of *tetA*, *qnrS*, *bla*_*CTX-M*_ in AWW3. EP2, which employed 0.5 mL aliquot with DNeasy Blood and Tissue Kit, generated the highest concentrations of *bla*_*CTX-M*_ in AWW2, the highest concentrations of *ermB* and *bla*_*NDM-1*_ in AWW3, and the highest concentrations of *tetA*, *ermB*, *qnrS*, *bla*_*CTX-M*_, *bla*_*NDM-1*_ in AWW4. In contrast, the lowest concentration of *tetA* in AWW2 was obtained using EP9, and the lowest concentrations of *bla*_*CTX-M*_, *ermB* and *qnrS* in AWW2 were obtained using EP10. In AWW3, EP3 employing 1 mL aliquot with DNeasy Blood and Tissue Kit generated the lowest concentrations of *tetA*, *bla*_*CTX-M*_, *ermB* and *qnrS*. In AWW4, the lowest concentrations were obtained using EP8 (1.5 mL pre-spin, AllPrep PowerViral DNA/RNA Kit with PM1) for *tetA*, EP9 for *bla*_*CTX-M*_ and EP4 for *ermB* and *qnrS*.

### Variations in ARGs concentrations among extraction protocols and aircraft wastewater samples

No significant difference in individual ARG concentrations was observed among the 10 extractions except for *bla*_*CTX-M*_ (χ^2^(9) = 23.58, *p* < 0.01) (Supplementary Tables ST6 and ST7). Similarly, multiple comparisons revealed no significant difference between extraction protocols using a Dunn–Bonferroni post-hoc test (Supplementary Tables ST8). Significant differences were observed across all extraction protocols (χ^2^(9) = 57.69, *p* < 0.0001), when concentrations of *tetA*, *ermB*, *qnrS* and *bla*_*CTX-M*_ were pooled and collectively analyzed (Supplementary Tables ST7). The concentrations of four ARG targets obtained with EP1 (0.2 mL), EP2 (0.5 mL) using DNeasy Blood and Tissue Kit  and EP5 (0.2 mL, AllPrep PowerViral DNA/RNA Kit with PM1) were significantly higher than that with EP3, EP9 and EP10 (Supplementary Tables ST9). Meanwhile, EP1 generated significantly higher concentrations of ARGs than that in EP4 (1.5 mL, pre-spin, DNeasy Blood and Tissue Kit  and EP6 (0.5 mL, AllPrep PowerViral DNA/RNA Kit with PM1) showed significantly higher values than that in EP9.

## Discussion

Aircraft wastewater could serve as an important reservoir for antibiotic resistance and contribute to the importation and spread of ARGs across borders ([1]; Bivins et al. [6]. qPCR-based measurement of ARGs has proven to be sensitive and specific for accurate assessment of ARG prevalence in wastewater [10, 18]. Development of optimized concentration and extraction workflows is a prerequisite to achieve accurate qPCR quantification of ARGs [22]. However, the sensitivity of qPCR-based assays may be compromised due to different wastewater matrices and discrepancies may exist in the detection and quantification of ARGs using various concentration and extraction protocols [2, 19, 21]. Unlike municipal wastewater, due to the low flushing volumes and minimal inputs of other liquids, aircraft wastewater is a highly concentrated waste stream containing biological waste (urine and feces, etc.) and toilet paper [6]. Additionally, after landing and draining wastewater from the storage tanks, disinfectant, and deodorizer (also referred to as “blue juice”) are commonly used to rinse the tanks as a part of routine ground handling operations and in some cases, remain inside the tanks before the next flight [6]. The blue juice residues that remain may further interfere with the efficacy extraction of nucleic acid and qPCR quantification.

In this study, ARGs indicative of anthropogenic inputs (*tetA*) and clinical relevance (*ermB*, *qnrS*, *bla*_*CTX-M*_ and *bla*_*NDM-1*_) were selected to represent chromosome- and plasmid- borne resistance genes with a wide range of prevalences in wastewater [9, 27, 30, 34]. AWW1-AWW4 including one “light blue” sample which contained a mixture of aircraft wastewater and blue juice residues (AWW1) were screened for *tetA*, *ermB*, *qnrS*, *bla*_*CTX-M*_, *bla*_*NDM-1*_ using qPCR with different sample volumes, concentration, and extraction procedures. Among the five ARG targets, *bla*_*CTX-M*_ and *bla*_*NDM-1*_ exhibited the lowest prevalence among the four aircraft wastewater samples with no detection in AWW1 and relatively low abundance (< LOD 0 to 3.79 log_10_ GC/mL for *bla*_*NDM-1*_ and 4.63 to 6.77 log_10_ GC/mL for *bla*_*CTX-M*_) in AWW2 to AWW4. *Bla*_*CTX-M*_ and *bla*_*NDM-1*_ confer resistance to most β-lactam antibiotics, which are clinically and epidemiologically important mechanisms of resistance in *Enterobacteriacae* and have been primarily detected in clinical environments [23, 25]. The import of multi-drug resistant *Enterobacteriacae* has been found to be carried over by international travelers and may pose a high risk of onward transmission and colonization across borders [1, 32]. For the rest of the ARG targets, a 100% detection rate was observed among aircraft wastewater samples except for AWW1 (light blue sample), indicating that aircraft wastewater sample treatment with blue juice might result in compromised detection and quantification of ARGs.

By collectively analyzing the concentrations of *tetA*, *ermB*, *qnrS* and *bla*_*CTX-M*_ obtained from AWW2 to AWW4, EP1, EP2 and EP5 using 0.2/0.5 mL sample volume with no pre-spin step yielded significantly greater ARG concentrations than in EP3 and EP9 using 1.0 mL and EP6 using 0.5 mL sample volume. The superior performance of small sample volumes for nucleic acid extraction has also been observed in previous studies [7, 22, 28]. On the one hand, employing small sample volume may minimize the interference of toilet paper during the extraction process, which may introduce PCR inhibitors and compromise amplification efficiency [11]. However, increasing the sample volume may overload the column of the two kits and lead to significantly lower yields than expected, as per the manufacturer’s instructions. Therefore, when extracting larger sample volumes, additional solids separation steps before or after extraction may be necessary to efficiently remove the toilet paper. This is especially crucial when employing chemical lysis with DNeasy Blood and Tissue Kit, as increased sample volumes (EP2 to EP4) could introduce extra amount of toilet paper that might not be readily digested (through our visual inspection) and require further centrifugation. However, for low abundance *bla*_*CTX-M*_ and *bla*_*NDM-1*_, smaller sample volumes may not be sufficient to guarantee sensitive detection in aircraft wastewater samples.

Among the extraction methods evaluated in this study, the highest concentrations of five ARG targets were primarily obtained with 0.2 and 0.5 mL volumes extracted with the DNeasy Blood and Tissue Kit, indicating that this kit might be more efficient for extraction from aircraft wastewater samples. Although no significant difference in ARG concentrations was observed between extraction protocols employing two different extraction kits with the same sample volumes, consistently low detection rates and concentrations of *tetA*, *ermB*, *qnrS* and *bla*_*CTX-M*_ were obtained using the AllPrep PowerViral DNA/RNA Kit with PM1 + TRIzol for lysis. TRIzol has been commonly used for nucleic acid extraction due to its ability to effectively denatures proteins and separate cellular components (DNA, RNA and proteins) based on their distinct chemical properties. Previous studies have demonstrated that DNA extractions conducted after RNA isolation using TRIzol buffers could generate low-quality DNA, and the combination of PM1 + TRIzol in the cell lysis step of Qiagen RNeasy PowerWater kit (Cat. No. 14700-50-NF, Qiagen) did not guarantee a higher DNA yield when compared with PM1 used alone [3, 31]. Furthermore, qPCR inhibition was observed in AWW1 and AWW2 when using extraction method EP9 and EP10. A possible explanation for these observations is the interaction between TRIzol with phenolic compounds, polysaccharides and other complex substances introduced by toilet paper [12]. Thus, the incorporation of TRIzol in DNA extraction may require specific protocols depending on sample types and downstream applications.

In conclusion, a small sample volume (down to 0.2 mL) may be sufficient for characterization of ARGs in concentrated aircraft wastewater using qPCR. While efficient removal of toilet paper presented in aircraft wastewater should be carefully considered without compromising the concentration and extraction efficiency of nucleic acid. Based on our preliminary results, aircraft wastewater monitoring of ARGs showed promising prospects and need to be further investigated regarding the import and spread of unique ARGs through air transport.

### Supplementary Information

Additional file 1.
