# Supplementary material for: Assessment of nucleic acid extraction protocols for antibiotic resistance genes (ARGs) quantification in aircraft wastewater
Source: Hum Genomics. 2024 May 30;18:54. doi: 10.1186/s40246-024-00617-5 (PMC11138010; doi:10.1186/s40246-024-00617-5)
Supplement: Supplementary file 1 — Additional file 1. [file 40246_2024_617_MOESM1_ESM.docx]

**Supplementary materials: Assessment of Nucleic Acid Extraction Protocols for Antibiotic Resistance Genes (ARGs) Quantification in Aircraft Wastewater**

Wendy J.M. Smith^a^, Yawen Liu^a,b^, Stuart L. Simpson^a^, Aaron Bivins^c^, Warish Ahmed^a,^*

^a^CSIRO Environment, Ecosciences Precinct, 41 Boggo Road, Dutton Park, QLD 4102, Australia

^b^State Key Laboratory of Marine Environmental Science, College of the Environment & Ecology, Xiamen University, Xiamen 361102, China

^c^Department of Civil & Environmental Engineering, Louisiana State University, Baton Rouge, LA 70803, United States of America

**Running title:** ARGs quantification approaches in aircraft wastewater

***Corresponding author.** Warish Ahmed. Mailing address: Ecosciences Precinct, 41 Boggo Road, Dutton Park 4102, Queensland, Australia. Tel.: +617 3833 5582; E-mail address: [Warish.Ahmed@csiro.au](mailto:Warish.Ahmed@csiro.au)

**Supplementary Table ST1**

Extraction protocols used in this study.

| Extraction protocols | Extraction kits used | Start aliquot vol. (mL) | Pre-spin | Vol extracted (mL) | Spin | Process | Lysis buffer | Homogenization | Extra spin after addition of proteinase K |  | Elution (200 μL) |
| --- | --- | --- | --- | --- | --- | --- | --- | --- | --- | --- | --- |
| EP1 | Qiagen Blood & Tissue | 0.2 | No | 0.2 | 21,000 g for 3 min | Pellet | 180 µL ALT + 20 µL proteinase K | 56°C 60 min | No | Follow manufacturers protocols | AE |
| EP2 | Qiagen Blood & Tissue | 0.5 | No | 0.5 | 21,000 g for 3 min | Pellet | 180 µL ALT + 20 µL proteinase K | 56°C 60 min | 1500 g for 30 s |  | AE |
| EP3 | Qiagen Blood & Tissue | 1 | No | 1 | 21,000 g for 3 min | Pellet | 180 µL ALT + 20 µL proteinase K | 56°C 60 min | 1500 g for 30 s |  | AE |
| EP4 | Qiagen Blood & Tissue | 1.5 | 1500 g for 30 s | 1 | 21,000 g for 3 min | Pellet | 180 µL ALT + 20 µL proteinase K | 56°C 60 min | No |  | AE |
| EP5 | Qiagen Power Viral DNA/RNA | 0.2 | No | 0.2 | 21,000 g for 3 min | Pellet | 800 µL PM1 | 10,000 rpm, 3 x 15s at 10s intervals | N/A |  | H_2_O |
| EP6 | Qiagen Power Viral DNA/RNA | 0.5 | No | 0.5 | 21,000 g for 3 min | Pellet | 800 µL PM1 | 10,000 rpm, 3 x 15s at 10s intervals | N/A |  | H_2_O |
| EP7 | Qiagen Power Viral DNA/RNA | 1 | No | 1 | 21,000 g for 3 min | Pellet | 800 µL PM1 | 10,000 rpm, 3 x 15s at 10s intervals | N/A |  | H_2_O |
| EP8 | Qiagen Power Viral DNA/RNA | 1.5 | 1500 g for 30 s | 1 | 21,000 g for 3 min | Pellet | 800 µL PM1 | 10,000 rpm, 3 x 15s at 10s intervals | N/A |  | H_2_O |
| EP9 | Qiagen Power Viral DNA/RNA | 1 | No | 1 | 21,000 g for 3 min | Pellet | 650 µL PM1 + 150 µL Trizol | 10,000 rpm, 3 x 15s at 10s intervals | N/A |  | H_2_O |
| EP10 | Qiagen Power Viral DNA/RNA | 1 | No | 1 | 21,000 g for 3 min | Pellet | 650 µL PM1 + 150 µL Trizol | 3200 rpm for 5 min (vortex) | N/A |  | H_2_O |

**Supplementary Table ST2**

Taman probes, primers and cycling parameters used in this study.

| ARGs | Sequences (5’-3’) | | Base pairs | Primers/probes (nM) | Cycling parameters | Reference |
| --- | --- | --- | --- | --- | --- | --- |
| *tetA* | Forward | TCAATTTCCTGACGGGCTG | 91 | 1000 | 10 min at 95℃, 40 cycles of 15 s at 95℃, 1 min at 60℃ | Borjesson et al., 2009 |
|  | Reverse | GAAGCGAGCGGGTTGAGAG |  | 1000 |  |  |
|  | Probe | AGTCGCACAAAGGCGAACGC |  | 100 |  |  |
| *ermB* | Forward | GATACCGTTTACGAAATTGG | 346 | 300 | 10 min at 95℃, 45 cycles of 15 s at 95℃,30 s at 60 ℃, 30 s at 72℃ | Chen et al., 2007 |
|  | Reverse | GAATCGAGACTTGAGTGTGC |  | 300 |  |  |
|  | Probe | AGCCAGTTTCGTCGTTAAATGCCC |  | 100 |  |  |
| *qnrS* | Forward | CGACGTGCTAACTTGCGTGA | 118 | 300 | 10 min at 95 ℃, 45 cycles of 15 s at 95℃,60 s at 60 ℃ | Colomer-Lluch et al., 2014 |
|  | Reverse | GGCATTGTTGGAAACTTGCA |  | 300 |  |  |
|  | Probe | AGTTCATTGAACAGGGTGA |  | 200 |  |  |
| *bla_CTX-M_* | Forward | ACCAACGATATCGCGGTGAT | 101 | 1000 | 10 min at 95℃, 45 cycles of 15 s at 95 ℃, 60 s at 60 ℃ | Colomer-Lluch et al., 2011 |
|  | Reverse | ACATCGCGACGGCTTTCT |  | 1000 |  |  |
|  | Probe | TCGTGCGCCGCTG |  | 200 |  |  |
| *bla_NDM-1_* | Forward | ATTAGCCGCTGCATTGAT | 154 | 500 | 10 min at 95 ℃, 45 cycles of 15 s at 95 ℃, 60 s at 60 ℃ | Ahammad et al., 2014 |
|  | Reverse | CATGTCGAGATAGGAAGTG |  | 500 |  |  |
|  | Probe | AGCAAATGGAAACTGGCGACCAAC |  | 100 |  |  |

**Supplementary Table ST3**

Concentration (ng/μL) of DNA and absorbance (*A*_260/280_) values for aircraft wastewater samples extracted using 10 extraction protocols.

| Extraction protocols | DNA concentrations (ng/μL) | *A*_260/280_ |
| --- | --- | --- |
| AWW1 |  |  |
| EP1 | 2.97 | 1.83 |
| EP2 | 2.13 | 1.56 |
| EP3 | 9.56 | 1.64 |
| EP4 | 2.03 | 1.39 |
| EP5 | 2.05 | 1.25 |
| EP6 | 3.10 | 1.35 |
| EP7 | 2.25 | 1.18 |
| EP8 | 3.78 | 1.07 |
| EP9 | 11.4 | 1.36 |
| EP10 | 18.3 | 1.4 |
| AWW2 |  |  |
| EP1 | 5.00 | 1.8 |
| EP2 | 5.38 | 1.87 |
| EP3 | 13.7 | 1.37 |
| EP4 | 8.51 | 1.65 |
| EP5 | 2.55 | 1.21 |
| EP6 | 4.38 | 1.6 |
| EP7 | 6.54 | 1.93 |
| EP8 | 3.29 | 1.89 |
| EP9 | 12.1 | 1.53 |
| EP10 | 24.8 | 1.16 |
| AWW3 |  |  |
| EP1 | 9.25 | 1.58 |
| EP2 | 10.7 | 1.50 |
| EP3 | 9.31 | 1.67 |
| EP4 | 11.4 | 1.33 |
| EP5 | 2.51 | 1.57 |
| EP6 | 4.52 | 1.63 |
| EP7 | 8.22 | 1.75 |
| EP8 | 8.67 | 1.57 |
| EP9 | 8.59 | 2.2 |
| EP10 | 8.38 | 1.61 |
| AWW4 | 5.66 | 1.07 |
| EP1 | 16.2 | 1.62 |
| EP2 | 11.1 | 1.77 |
| EP3 | 9.23 | 1.15 |
| EP4 | 2.04 | 1.71 |
| EP5 | 4.51 | 1.78 |
| EP6 | 9.67 | 1.63 |
| EP7 | 6.69 | 1.61 |
| EP8 | 9.14 | 1.60 |
| EP9 | 16.1 | 1.59 |
| EP10 | 5.66 | 1.07 |

**Supplementary Table ST4**

qPCR performance characteristics of *tetA*, *ermB*, *qnrS*, *bla_CTX-M_* and *bla_NDM-1_* assays.

| Assays | qPCR performance characteristics | | | |
| --- | --- | --- | --- | --- |
|  | Efficiency (%) | Linearity (r^2^) | Slope | Y-intercept |
| *tetA* | 92.8 | 0.994 | -3.51 | 39.8 |
| *ermB* | 97.7 | 0.997 | -3.38 | 38.8 |
| *qnrS* | 110 | 0.940 | -3.10 | 41.8 |
| *bla_CTX-M_* | 91.3 | 0.953 | -3.55 | 45.9 |
| *bla_NDM-1_* | 90.1 | 1.00 | -3.58 | 40.5 |

**Supplementary Table ST5**

The mean log_10_ gene copies (GC)/mL of ARGs in aircraft wastewater samples.

| ARGs | Methods | Mean ± SD log_10_ GC/mL | | | |
| --- | --- | --- | --- | --- | --- |
|  |  | AWW1 | AWW2 | AWW3 | AWW4 |
| *tetA* | EP1 | 3.45 ± 0.18 | 6.19 ± 0.01 | 6.10 ± 0.02 | 5.95 ± 0.02 |
|  | EP2 | 2.90 ± 0.34 | 5.89 ± 0.06 | 6.03 ± 0.00 | 6.34 ± 0.01 |
|  | EP3 | 3.29 ± 0.01 | 5.83 ± 0.04 | 5.44 ± 0.02 | 5.72 ± 0.05 |
|  | EP4 | 3.07 ± 0.07 | 5.87 ± 0.03 | 5.76 ± 0.01 | 5.63 ± 0.04 |
|  | EP5 | 0 | 5.95 ± 0.01 | 5.92 ± 0.01 | 5.93 ± 0.01 |
|  | EP6 | 0 | 5.99 ± 0.02 | 5.86 ± 0.00 | 5.80 ± 0.02 |
|  | EP7 | 2.77 ± 0.34 | 5.68 ± 0.02 | 5.75 ± 0.01 | 5.77 ± 0.01 |
|  | EP8 | 2.52 ± 0.01 | 5.85 ± 0.02 | 5.94 ± 0.03 | 5.59 ± 0.04 |
|  | EP9 | 0 | 5.30 ± 0.00 | 5.51 ± 0.04 | 5.66 ± 0.03 |
|  | EP10 | 0 | 5.31 ± 0.07 | 5.65 ± 0.00 | 5.91 ± 0.04 |
| *ermB* | EP1 | 3.30 ± 0.28 | 8.41 ± 0.03 | 8.07 ± 0.01 | 6.94 ± 0.13 |
|  | EP2 | 2.92 ± 0.47 | 7.96 ± 0.01 | 8.24 ± 0.03 | 8.34 ± 0.02 |
|  | EP3 | 3.78 ± 0.02 | 7.78 ± 0.04 | 6.73 ± 0.02 | 6.71 ± 0.04 |
|  | EP4 | 3.67 ± 0.14 | 8.08 ± 0.03 | 7.90 ± 0.01 | 6.60 ± 0.08 |
|  | EP5 | 0 | 8.31 ± 0.03 | 7.80 ± 0.02 | 7.51 ± 0.04 |
|  | EP6 | 2.62 ± 0.31 | 8.41 ± 0.05 | 7.86 ± 0.03 | 7.41 ± 0.04 |
|  | EP7 | 3.03 ± 0.22 | 8.11 ± 0.01 | 7.84 ± 0.02 | 7.39 ± 0.07 |
|  | EP8 | 3.07 ± 0.22 | 8.20 ± 0.02 | 8.10 ± 0.12 | 6.95 ± 0.09 |
|  | EP9 | 0 | 8.04 ± 0.02 | 7.60 ± 0.07 | 7.28 ± 0.09 |
|  | EP10 | 0 | 6.58 ± 1.00 | 7.67 ± 0.02 | 7.62 ± 0.25 |
| *qnrS* | EP1 | 3.84 ± 0.04 | 6.56 ± 0.13 | 6.80 ± 0.02 | 5.10 ± 0.05 |
|  | EP2 | 0 | 6.22 ± 0.03 | 6.37 ± 0.04 | 6.40 ± 0.03 |
|  | EP3 | 3.43 ± 0.04 | 6.45 ± 0.10 | 4.95 ± 0.09 | 4.93 ± 0.10 |
|  | EP4 | 3.14 ± 0.41 | 6.25 ± 0.08 | 6.41 ± 0.03 | 4.92 ± 0.08 |
|  | EP5 | 0 | 6.26 ± 0.05 | 6.47 ± 0.09 | 5.44 ± 0.11 |
|  | EP6 | 0 | 6.20 ± 0.04 | 6.56 ± 0.11 | 5.23 ± 0.06 |
|  | EP7 | 0 | 5.94 ± 0.02 | 6.48 ± 0.05 | 5.17 ± 0.04 |
|  | EP8 | 0 | 6.29 ± 0.02 | 6.69 ± 0.01 | 5.10 ± 0.02 |
|  | EP9 | 0 | 5.88 ± 0.02 | 6.11 ± 0.05 | 4.98 ± 0.04 |
|  | EP10 | 0 | 5.73 ± 0.10 | 6.16 ± 0.08 | 4.93 ± 0.08 |
| *bla_CTX-M_* | EP1 | 0 | 6.74 ± 0.03 | 6.66 ± 0.03 | 5.18 ± 0.19 |
|  | EP2 | 0 | 6.76 ± 0.01 | 6.57 ± 0.01 | 6.77 ± 0.02 |
|  | EP3 | 0 | 6.31 ± 0.03 | 4.63 ± 0.23 | 4.95 ± 0.07 |
|  | EP4 | 0 | 6.36 ± 0.03 | 6.22 ± 0.02 | 4.88 ± 0.07 |
|  | EP5 | 0 | 6.53 ± 0.07 | 6.44 ± 0.04 | 5.31 ± 0.13 |
|  | EP6 | 0 | 6.58 ± 0.05 | 6.32 ± 0.05 | 5.06 ± 0.24 |
|  | EP7 | 0 | 6.06 ± 0.01 | 6.17 ± 0.01 | 4.84 ± 0.21 |
|  | EP8 | 0 | 6.38 ± 0.05 | 6.38 ± 0.01 | 4.91 ± 0.05 |
|  | EP9 | 0 | 6.01 ± 0.04 | 5.82 ± 0.07 | 4.78 ± 0.07 |
|  | EP10 | 0 | 5.96 ± 0.09 | 5.98 ± 0.03 | 4.90 ± 0.12 |
| *bla_NDM-1_* | EP1 | 0 | 3.79 ± 0.11 | 0 | 0 |
|  | EP2 | 0 | 3.41 ± 0.02 | 3.58 ± 0.06 | 3.39 ± 0.28 |
|  | EP3 | 0 | 0 | 0 | 0 |
|  | EP4 | 0 | 3.40 ± 0.10 | 0 | 0 |
|  | EP5 | 0 | 3.35 ± 0.30 | 0 | 0 |
|  | EP6 | 0 | 3.52 ± 0.17 | 2.96 ± 0.22 | 0 |
|  | EP7 | 0 | 2.97 ± 0.21 | 0 | 2.58 ± 0.10 |
|  | EP8 | 0 | 3.41 ± 0.22 | 0 | 0 |
|  | EP9 | 0 | 0 | 0 | 0 |
|  | EP10 | 0 | 0 | 0 | 0 |

**Supplementary Table ST6**

Repeated measure ANOVA comparing the performance of different methods on mean log_10_ GC/mL of *tetA* and *bla_NDM-1_* in all aircraft wastewater samples.

| ARGs | Assume sphericity? | F | *P* value | *P* value summary | Geisser-Greenhouse’s epilon | R^2^ |
| --- | --- | --- | --- | --- | --- | --- |
| *tetA* | No | 3.569 | 0.1615 | Not significant | 0.1633 | 0.641 |
| *bla_NDM-1_* | No | 1.902 | 0.2629 | Not significant | 0.3327 | 0.487 |

**Supplementary Table ST7**

Friedman test comparing the performance of different extraction protocols on mean log_10_ GC/mL of *ermB*, *qnrS*, *bla_CTX-M_* and ARGs (*tetA*, *ermB*, *qnrS* and *bla_CTX-M_*) in all aircraft wastewater samples.

| ARGs | Friedman statistics | *P* value | Are means significantly different? |
| --- | --- | --- | --- |
| *ermB* | 11.07 | 0.2708 | No |
| *qnrS* | 13.76 | 0.1310 | No |
| *bla_CTX-M_* | 23.58 | <0.01 | Yes |
| ARGs (*tetA*, *ermB*, *qnrS* and *bla_CTX-M_*) | 57.69 | <0.0001 | Yes |

**Supplementary Table ST8**

Dunn’s multiple comparisons test of extraction protocols performance on mean log_10_ GC/mL of *bla_CTX-M_* in all aircraft wastewater samples.

| Dunn's multiple comparisons test | Rank sum diff. | Significant? | Adjusted P Value |
| --- | --- | --- | --- |
| EP1 vs. EP2 | -2.000 | No | >0.9999 |
| EP1 vs. EP3 | 16.00 | No | >0.9999 |
| EP1 vs. EP4 | 14.00 | No | >0.9999 |
| EP1 vs. EP5 | 3.000 | No | >0.9999 |
| EP1 vs. EP6 | 6.000 | No | >0.9999 |
| EP1 vs. EP7 | 18.00 | No | 0.6849 |
| EP1 vs. EP8 | 9.000 | No | >0.9999 |
| EP1 vs. EP9 | 22.00 | No | 0.1356 |
| EP1 vs. EP10 | 19.00 | No | 0.4684 |
| EP2 vs. EP3 | 18.00 | No | 0.6849 |
| EP2 vs. EP4 | 16.00 | No | >0.9999 |
| EP2 vs. EP5 | 5.000 | No | >0.9999 |
| EP2 vs. EP6 | 8.000 | No | >0.9999 |
| EP2 vs. EP7 | 20.00 | No | 0.3150 |
| EP2 vs. EP8 | 11.00 | No | >0.9999 |
| EP2 vs. EP9 | 24.00 | No | 0.0545 |
| EP2 vs. EP10 | 21.00 | No | 0.2084 |
| EP3 vs. EP4 | -2.000 | No | >0.9999 |
| EP3 vs. EP5 | -13.00 | No | >0.9999 |
| EP3 vs. EP6 | -10.00 | No | >0.9999 |
| EP3 vs. EP7 | 2.000 | No | >0.9999 |
| EP3 vs. EP8 | -7.000 | No | >0.9999 |
| EP3 vs. EP9 | 6.000 | No | >0.9999 |
| EP3 vs. EP10 | 3.000 | No | >0.9999 |
| EP4 vs. EP5 | -11.00 | No | >0.9999 |
| EP4 vs. EP6 | -8.000 | No | >0.9999 |
| EP4 vs. EP7 | 4.000 | No | >0.9999 |
| EP4 vs. EP8 | -5.000 | No | >0.9999 |
| EP4 vs. EP9 | 8.000 | No | >0.9999 |
| EP4 vs. EP10 | 5.000 | No | >0.9999 |
| EP5 vs. EP6 | 3.000 | No | >0.9999 |
| EP5 vs. EP7 | 15.00 | No | >0.9999 |
| EP5 vs. EP8 | 6.000 | No | >0.9999 |
| EP5 vs. EP9 | 19.00 | No | 0.4684 |
| EP5 vs. EP10 | 16.00 | No | >0.9999 |
| EP6 vs. EP7 | 12.00 | No | >0.9999 |
| EP6 vs. EP8 | 3.000 | No | >0.9999 |
| EP6 vs. EP9 | 16.00 | No | >0.9999 |
| EP6 vs. EP10 | 13.00 | No | >0.9999 |
| EP7 vs. EP8 | -9.000 | No | >0.9999 |
| EP7 vs. EP9 | 4.000 | No | >0.9999 |
| EP7 vs. EP10 | 1.000 | No | >0.9999 |
| EP8 vs. EP9 | 13.00 | No | >0.9999 |
| EP8 vs. EP10 | 10.00 | No | >0.9999 |
| EP9 vs. EP10 | -3.000 | No | >0.9999 |

**Supplementary Table ST9**

Dunn’s multiple comparisons test of method performance on mean log_10_ GC/mL of ARGs (*tetA*, *ermB*, *qnrS* and *bla_CTX-M_*) in all aircraft wastewater samples.

| Dunn's multiple comparisons test | Rank sum diff. | Significant? | Adjusted P Value |
| --- | --- | --- | --- |
| EP1 vs. EP2 | 5.000 | No | >0.9999 |
| EP1 vs. EP3 | 64.00 | Yes | 0.0007 |
| EP1 vs. EP4 | 51.00 | Yes | 0.0263 |
| EP1 vs. EP5 | 13.00 | No | >0.9999 |
| EP1 vs. EP6 | 18.00 | No | >0.9999 |
| EP1 vs. EP7 | 47.00 | No | 0.0689 |
| EP1 vs. EP8 | 27.00 | No | >0.9999 |
| EP1 vs. EP9 | 72.00 | Yes | <0.0001 |
| EP1 vs. EP10 | 63.00 | Yes | 0.0010 |
| EP2 vs. EP3 | 59.00 | Yes | 0.0031 |
| EP2 vs. EP4 | 46.00 | No | 0.0867 |
| EP2 vs. EP5 | 8.000 | No | >0.9999 |
| EP2 vs. EP6 | 13.00 | No | >0.9999 |
| EP2 vs. EP7 | 42.00 | No | 0.2084 |
| EP2 vs. EP8 | 22.00 | No | >0.9999 |
| EP2 vs. EP9 | 67.00 | Yes | 0.0003 |
| EP2 vs. EP10 | 58.00 | Yes | 0.0041 |
| EP3 vs. EP4 | -13.00 | No | >0.9999 |
| EP3 vs. EP5 | -51.00 | Yes | 0.0263 |
| EP3 vs. EP6 | -46.00 | No | 0.0867 |
| EP3 vs. EP7 | -17.00 | No | >0.9999 |
| EP3 vs. EP8 | -37.00 | No | 0.5675 |
| EP3 vs. EP9 | 8.000 | No | >0.9999 |
| EP3 vs. EP10 | -1.000 | No | >0.9999 |
| EP4 vs. EP5 | -38.00 | No | 0.4684 |
| EP4 vs. EP6 | -33.00 | No | >0.9999 |
| EP4 vs. EP7 | -4.000 | No | >0.9999 |
| EP4 vs. EP8 | -24.00 | No | >0.9999 |
| EP4 vs. EP9 | 21.00 | No | >0.9999 |
| EP4 vs. EP10 | 12.00 | No | >0.9999 |
| EP5 vs. EP6 | 5.000 | No | >0.9999 |
| EP5 vs. EP7 | 34.00 | No | 0.9850 |
| EP5 vs. EP8 | 14.00 | No | >0.9999 |
| EP5 vs. EP9 | 59.00 | Yes | 0.0031 |
| EP5 vs. EP10 | 50.00 | Yes | 0.0337 |
| EP6 vs. EP7 | 29.00 | No | >0.9999 |
| EP6 vs. EP8 | 9.000 | No | >0.9999 |
| EP6 vs. EP9 | 54.00 | Yes | 0.0122 |
| EP6 vs. EP10 | 45.00 | No | 0.1086 |
| EP7 vs. EP8 | -20.00 | No | >0.9999 |
| EP7 vs. EP9 | 25.00 | No | >0.9999 |
| EP7 vs. EP10 | 16.00 | No | >0.9999 |
| EP8 vs. EP9 | 45.00 | No | 0.1086 |
| EP8 vs. EP10 | 36.00 | No | 0.6849 |
| EP9 vs. EP10 | -9.000 | No | >0.9999 |

**References**

Ahammad, Z.S., Sreekrishnan, T.R., Hands, C.L., Knapp, C.W., Graham, D.W., 2014. Increased waterborne blaNDM-1 resistance gene abundances associated with seasonal human pilgrimages to the upper Ganges River. Environ. Sci. Technol. 48, 3014–3020.

Borjesson, S., Dienues, O., Jarnheimer, P.A., Olsen, B., Matussek, A., Lindgren, P.E., 2009. Quantification of genes encoding resistance to aminoglycosides, beta-lactams and tetracyclines in wastewater environments by real-time PCR. Int. J. Environ. Health Res. 19, 219–230.

Chen, J., Yu, Z., Michel, F.C., Jr. Wittum, T., Morrison, M., 2007. Development and application of real-time PCR assays for quantification of erm genes conferring resistance to macrolides-lincosamides-streptogramin B in livestock manure and manure management systems. Appl. Environ. Microbiol. 73, 4407–4416.

Colomer-Lluch, M., Jofre, J, Muniesa, M., 2011. Antibiotic resistance genes in the bacteriophage DNA fraction of environmental samples. PloS one. 6, e17549.

Colomer-Lluch, M., Jofre, J., Muniesa, M., 2014. Quinolone resistance genes (qnrA and qnrS) in bacteriophage particles from wastewater samples and the effect of inducing agents on packaged antibiotic resistance genes. J. Antimicrob Chemother. 69, 1265–1274.
